# Current issues of destination therapy in Japan: to achieve 5−year or even longer survival

**DOI:** 10.1007/s10047-024-01458-8

**Published:** 2024-07-09

**Authors:** Masahiko Ando, Minoru Ono

**Affiliations:** Department of Cardiovascular Surgery, The University of Tokyo Hospital, Hongo 7-3-1, Bunkyo, Tokyo, 113-8655 Japan

**Keywords:** Destination therapy, Ventricular assist device, Heart failure

## Abstract

In April 2021, destination therapy (DT) was finally approved in Japan. Since DT does not aim at heart transplantation (HT), our goal is to have a patient safely remain on an implantable ventricular assist device (VAD) for the rest of his/her life. To achieve this goal, similarly to bridge-to-transplant (BTT) patients, the authors believe the following six aspects are even more crucial in DT patients: (1) to appropriately assess risks before implantation, (2) to carefully determine the ability to manage the device by multidisciplinary discussions, (3) to prevent complications by improving the quality of care, (4) to expand the number of facilities that can take care of DT patients by improving collaboration among the facilities, (5) to reduce the burden of caregivers by utilizing social resources, and (6) to establish a home palliative care system based on advance care planning. In addition, for elderly DT patients to live happy and long lives, it is essential to help them to find a purpose of life and to keep activities of daily living, such as employment, schooling, and participation in social activities, just like the general elderly population. Our goals are not only to do our best for the patients just in front of us, but also to establish a system to follow up our DT cohort, same as BTT one, by all-Japan manner. In the present review, we discuss the current state of DT in Japan and what we need to focus on to maintain or improve its long-term performance.

## Background

In April 2021, destination therapy (DT) was finally approved in Japan. Since DT does not aim at heart transplantation (HT), one of the goals is to have a patient remain on an implantable ventricular assist device (VAD) for the rest of his/her life. However, in Japan, where waiting time for HT is extremely long, we have been following bridge-to-transplant (BTT) patients with a nuance very close to the same goal of what DT aims for. For example, BTT patients who are over 60 years old at the time of HT registration are almost never actually getting HT in terms of the priority of donor heart allocation. They are an unique population of BTT patients in our country, called “actually DT”.

**Fig. 1:**
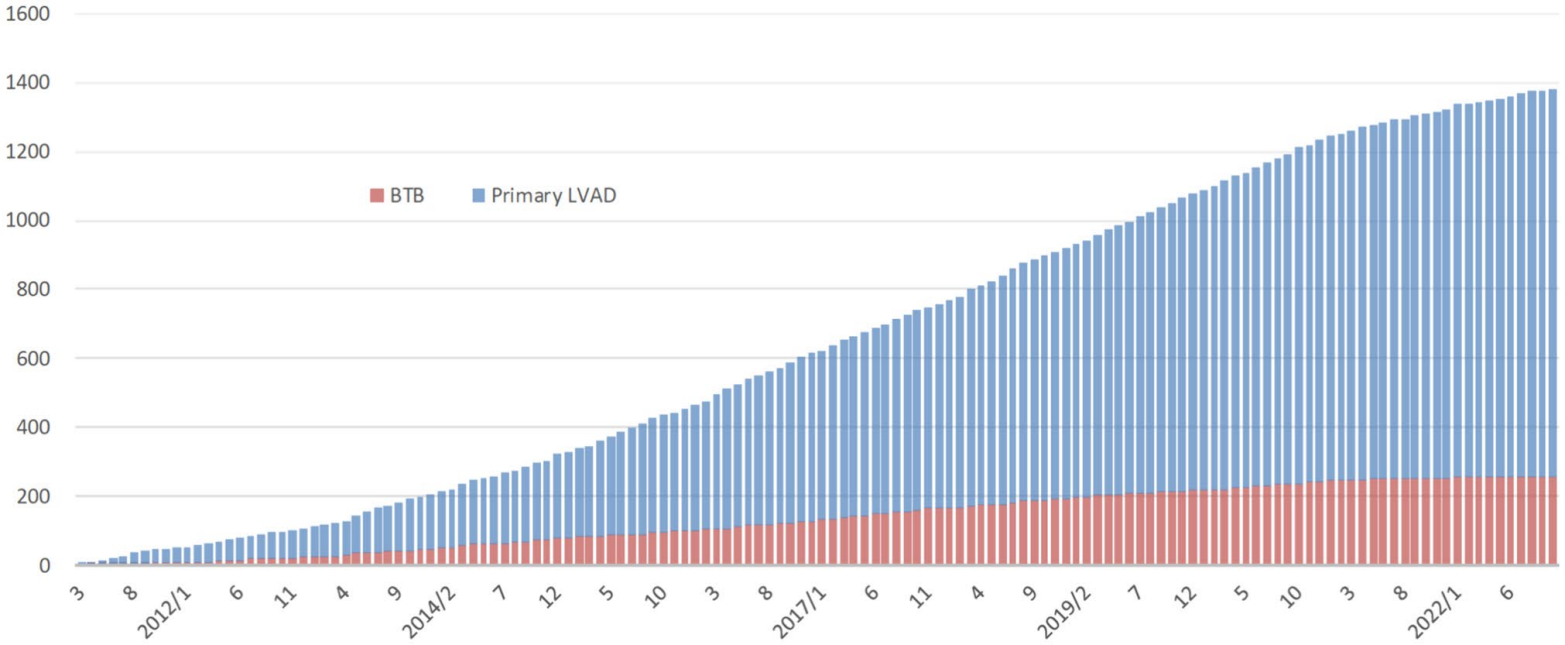
Number of newly implanted implantable VADs per year [1]. The number of BTT implantable VADs implanted is increasing dramatically each year, and the number of DT implantable VADs has been added since May 2021.

According to the “Japanese Registry for Mechanically Assisted Circulatory Support (J-MACS)”, approximately 1400 implantable VAD procedures have been performed in Japan as of December 31, 2022 (Fig. 1), and the number of cases continues to increase [1]. Along with the annual number of HT has been from 80 to 120 for the past several years [2], the waiting time for BTT candidates to be registered recently as status 1 is expected to be 6 to 8 years, which is quite similar to the DT situation in Western countries. Additionally, not a few BTT cases are getting ineligible for HT (status 3) and consequently becoming “DT” for chronic renal failure, respiratory failure, or cerebrovascular accidents during such a long waiting period. The outcomes of HT candidates in Japan are demonstrated on the website by Japan Organ Transplant Network [3]. In fact, as of February 2024, among the total number of 2403 candidates registered for HT, 582 patients (24.2%) deceased before HT, and some of them became ineligible for HT for the fore-mentioned VAD-associated complications. Moreover, among 860 HT candidates on the waitlist as of February 2024, 34 patients (4.0%) are status 3 [3]. For this reason, for many years in Japan, even before DT was approved in 2021, we had been doing our very best to find ways to help “BTT” patients permanently live well on their implantable VADs, making the outcomes the best in the world.

The authors believe that both local medical regions and the country should establish a system that can safely manage long-term support for 5 years, or even more, for the increasing number of DT candidates in the future. Not to mention, especially for DT patients and their caregivers, it is necessary to consider not only their safety issues but also their quality of life (QOL), who will spend the rest of their lives with implantable VAD. In this review, we discuss the current state of DT in Japan and what we need to focus on to maintain or improve its long-term performance.

## Miscellaneous patient features included in DT candidates in Japan

**Fig. 2:**
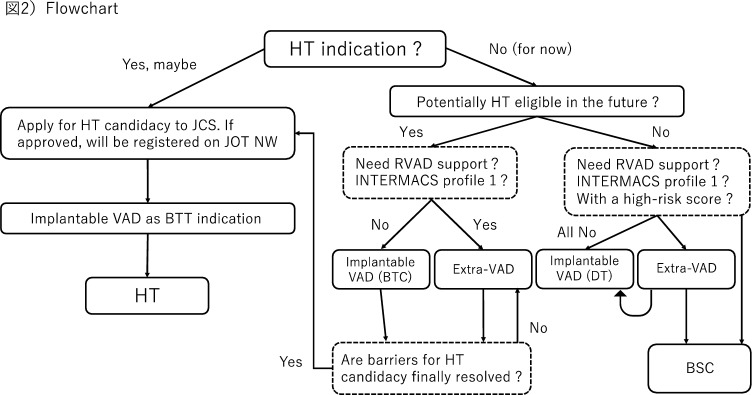
Flowchart of VAD therapy for patients with severe heart failure in Japan. *HT* heart transplantation, *VAD* ventricular assist device, *extra-VAD* extracorporeal VAD, *BTC* bridge to candidacy, *BTT* bridge to transplantation, *JCS* Japanese circulation society, *JOT NW* Japan organ transplant network, *RVAD* right VAD. HT indication is generally judged by Japanese Circulation Society to equalize the indication threshold among institutions, DT and BTC indication is determined only the home facilities.

There are various reasons why a DT candidate is not eligible for HT. In a true, or limited, definition of DT, for example, the patient cannot be registered for HT because of age (65 years old or older). On the other hand, within a broad definition of DT, bridge-to-candidacy (BTC) candidates are included. Although they are not eligible for HT at the time of implantable VAD implantation, they might finally become eligible if the barriers for HT registration is resolved, such as reversible renal dysfunction or high pulmonary vascular resistance. If they have a history of malignancy, first it should be completely cured by surgery or other treatment modality. Then for HT registration, the candidate needs to be free from adjunctive therapies, and discussed on multidisciplinary malignancy boards to obtain comments from specialists to demonstrate 5-year malignancy-free survival is more than 95%. Flow chart for DT/BTC managements is shown in Fig. 2. In DT/BTC cases, once such exclusion criteria for HT are resolved by implantable VAD support, they could be eventually registered for HT and they can continue implantable VAD support as BTT.

In the prior era, when DT was not yet approved, candidates with unstable hemodynamics who could not undergo HT registration tests such as gastrointestinal endoscopy or myocardial scintigraphy, needed to undergo IMPELLA ^®^ (Abiomed ^®^ Japan) or an extracorporeal VAD, proceed with a thorough examination for registration, complete HT registration, and finally undergo implantable VAD implantation as BTT. Thanks to the approval of DT in 2021, now it became possible for us to implant an implantable VAD as DT/BTC in candidates who have not yet been thoroughly evaluated for HT registration. This is quite helpful for them, because they can skip such risky procedures as IMPELLA ^®^ or extracorporeal VAD, if they fall within the scope of DT criteria. After implantable VAD implantation, probably the candidates could take routine check-up for HT registration, as needed, and if no exclusionary conditions are found, they could be finally listed for HT and their implantable VAD indication could be converted to BTT from DT/BTC. If some exclusionary conditions are found, unfortunately, they will remain in DT for the rest of their lives. Clearly, especially in candidates with unstable hemodynamics, such a strategy, first implanting an implantable VAD as DT/BTC and then deciding whether HT is indicated or not, makes more sense.

In fact, the INTERMACS registry in the United States (US) reported that 94.6% of new implantable VAD cases in 2021 were implanted as DT or BTC, and only about 5% were implanted as BTT [4]. In the US, BTT is decreasing, and DT and BTC are increasing. This tendency is brought to them, partly because they implemented a new HT allocation rule in 2018 [5]. Now, the candidates with temporary support devices are more likely to get HT, and HT centers prefer to use IMPELLA^®^ and IABP (intra-aortic balloon pumping) from the axillary artery as a bridge device to transplantation, instead of permanent implantable VAD, which is reasonable. While in Japan, implantable VAD with BTT is expected to be the mainstream for the time being.

## Six points for long-term safe assistance in DT cases

Based on the background above, in the present manuscript, the authors would like to discuss how to safely assist a typical true DT case for a long period of time. The typical DT candidate here is assumed to be a 70-year-old male with ischemic cardiomyopathy who is not eligible for HT, complicated with renal dysfunction with serum creatinine level of 2.6 mg/dl, but his serum albumin level is 2.8 g/dl, indicating relatively well-maintained nutritional status. In this case, the J-HMRS (Japan HeartMate Risk Score) [6, 7] is 1.82, which corresponds to medium risk in an experienced institution as defined by the J-HMRS. What kind of care is needed to ensure that patients like this case can live to 75 or 80 years of age while keeping their quality of life? Apparently, the tips for safe maintenance and management of DT cases would be the same as the ones for BTT cases, as mentioned above. In our opinion, the following six points are crucial.

(1) To appropriately assess risks before implantation, (2) to carefully determine the ability to manage the device by multidisciplinary discussions, (3) to prevent complications by improving the quality of care, (4) to expand the number of facilities that can take care of DT patients by improving collaboration among the facilities, (5) to reduce the burden of caregivers by utilizing social resources, and (6) to establish a home palliative care system based on advance care planning.

1. To appropriately assess risks before implantation.

As mentioned in the Background, the introduction of DT will surely further increase the number of implantable VAD implantation in Japan. However, it does not necessarily mean that we could save all the patients with severe heart failure who are not eligible for HT. Nearly thirty million Japanese Yen in medical costs are required to keep patients well for one year after DT-implantable VAD implantation. Since DT is covered by insurance, healthcare providers must consistently adhere the standard of patient selection and need to demonstrate clinical outcomes, commensurate with the medical costs invested based on public burden. Otherwise, the very existence of DT itself will be in jeopardy. Therefore, to continuously achieve long-term survival of 5 years or longer even in the future DT cases, it is essential to first carefully select patients who are candidates for DT. Although the criteria for DT are quite detailed [6, 7], the point is that to some extent, the decision must be made at the discretion of healthcare providers. It is noteworthy that while HT indication is generally judged by Japanese Circulation Society (Fig. 2) to equalize the indication threshold among institutions, DT and BTC indication is determined only the home facilities. This is probably why the background of DT population in Japan is quite heterogeneous.

The first bottleneck is that there is no correct model for predicting long-term prognosis in DT cases in Japan, making it difficult to determine the suitability for DT. In determining the suitability for DT in our country, the J-HMRS has been calculated based on age, serum albumin, serum creatinine, and PT-INR (prothrombin time, international normalized ratio), and if the patient is classified as medium- or high risk, the suitability for DT should be carefully determined taking the patient’s age into consideration. However, the J-HMRS is based on the HMRS (HeartMate II Risk Score) in the United States [8], and the base data for HMRS calculations are derived from HeartMate “II” clinical trials including “both BTT and DT” in the United States and Europe. Therefore, some are suspicious of the use of J-HMRS for prognostic prediction of DT using HeartMate3 in our country.

In the other hand, the HeartMate3 Risk Score, which is calculated based on age, previous open heart surgery, sodium level, BUN (blood urea nitrogen), left ventricular end-diastolic diameter, right atrial pressure-pulmonary artery wedge pressure ratio (RAP/PAWP ratio), etc., has been proposed as a risk score for HeartMate3 [9]. Because it was calculated based on data from the MOMENTUM3 clinical trial, it excludes patients with a serum creatinine level of 2.5 mg/dl or higher, a serum albumin level of less than 3 g/dl, and a PT-INR of 2.0 or higher, and does not reflect real-world population [10]. The prior sample case of a 70-year-old man was not even included in the construction of the prediction model, making it difficult to predict the long-term prognosis of DT cases in Japan with the HeartMate3 Risk Score. Recently, Imamura et al. reported “the J-MACS risk score” based on age, history of cardiac surgery, serum creatinine, and RAP/PAWP ratio, to estimate 3-year mortality rate after implantable VAD implantation using large-scale multicenter Japanese data. Hopefully in the future, the clinical utility of this scoring to guide the indication of DT will be clarified. Although the J-MACS risk score is introduced in the current DT criteria in our country, one of the major limitations of J-MACS risk score is, as they would admit, that they needed to establish the scoring system itself based on the historical devices that are not currently in use, especially for DT. Other issues include, for example, although there are no exclusion criteria for liver function other than cirrhosis (Table 1) [6, 7], the indication for DT should be judged carefully in patients with total bilirubin levels exceeding 5 mg/dl.

(2) To carefully determine the ability to manage the device by multidisciplinary discussions.

**Table 1:** Patient selection criteria for destination therapy [7]

Target patients	Diseases/etiologies	Advanced heart failure ineligible for heart transplantation Etiologies include dilated cardiomyopathy, dilated phase hypertrophic cardiomyopathy, ischemic heart disease, valvular disease, congenital heart disease, drug-induced cardiomyopathy, post-myocarditis, cardiac sarcoidosis, etc.
Inclusion criteria	New York Heart Association class	III–IV (with a history of class IV, in principle)
	Stage classification	D
	INTERMACS profile	2–4 (excluding high-risk or unstable profile 2 for those aged ≥ 65 years)
	Drug treatment	Guideline-directed medical therapy with diuretics, angiotensin-converting enzyme inhibitor/angiotensin II type 1 receptor blocker/angiotensin-neprilysin inhibitor, β-blocker, mineralocorticoid receptor antagonist, and sodium-glucose transporter 2 inhibitor (potassium/sodium hyperpolarization-activated cyclic nucleotide-gated channel 4 inhibitor if indicated) should be implemented
	Dependence on inotropes and mechanical circulatory support	Dependent on dobutamine, dopamine, norepinephrine, phosphodiesterase-3 inhibitors, etc., or on an intra-aortic balloon pump, catheter-based transaortic microaxial pumps, or paracorporeal VAD, etc.
	J-HeartMate Risk Score	Take into consideration to discuss indication
	Age	For those aged ≥ 65 years, hemodynamics, end-organ function, nutritional status or cognitive function should be examined more carefully
	Body surface area	Determined individually with each device system
	Functional status	Patients with significantly impaired quality of life, who cannot expect a survival benefit with other treatments but can expect improved quality of life for long-term at home and rehabilitation after receiving an implantable LVAD
	Comorbidities	≥ 5-year life expectancy determined based on comorbidities
	Caregivers	At least one caregiver who can live with the patient for about 6 months after the first discharge is necessary (desirably, caregivers or public services can continue to provide nursing care for longer than 6 months)
	Self-management ability	Those aged ≥ 65 years shall have a Mini-Mental State Examination score ≥ 24 points and a Trail Making Test-B score ≤ 300 s before surgery For those aged < 65 years, their self-management abilities are determined at the implant center before surgery. In all cases, sufficient self-management abilities shall be reconfirmed before discharge to plan the level of nursing care by a caregiver
	Understanding of treatment	Good adherence to medication, continued abstinence from alcohol and smoking, understanding of the limitations and complications of VAD therapy, and adequate support of family members in cooperation with the patient
	Understanding of end-of-life care	Patients and their family members understand and consent to the end-of-life care of DT
Exclusion criteria	Infection	Severe infection
	Respiratory disease	Pulmonary artery embolism that developed within 30 days
	Cardiovascular diseases	Early after cardiotomy
		High possibility of postoperative right heart failure
		Untreatable abdominal aneurysm or severe peripheral vascular disease
		Thoracic aortic aneurysm/ventricular aneurysm/ventricular septal perforation
		Untreatable moderate or severe aortic regurgitation
		Mechanical aortic valve that cannot be replaced with a biological valve
		Severe calcification in the thoracic aorta
	Psychiatric/neurologic disorders	Severe central nervous system disorders
		History of substance abuse or alcohol dependence
		Encephalopathy, psychiatric disorders or neuromuscular disorders that may preclude self-management of a device
	End-organ failure	Maintenance dialysis
		Liver cirrhosis
	Pregnancy	Being pregnant
	Other	Severe obesity, administration of immunosuppressive drugs other than low-dose steroids, administration of anticancer drugs, refusal of blood transfusion, or conditions that are considered as ineligible by the institutional committee

The second problem is how to assess the ability of DT candidates to manage the device. DT selection criteria state that “in patients 65 years of age or older, preoperative MMSE (mini mental state examination) score of 24 or more and TMT-B (trail making test—B) of 300 s or less should be confirmed” [6, 7]. However, even in candidates that meet these criteria, there are frequently cases in clinical practice in which patients have difficulty in learning device management guidance. It is preferable to determine the suitability of DT comprehensively in consultation with a clinical engineer or physical therapist. The situation is even more difficult when determining the suitability of patients under 65 years of age, since the criteria states that the decision should be made preoperatively at the implanting hospital. Because some DT candidates stay in the intensive care unit for long periods of time, assisted by IMPELLA^®^ and others, they are often complicated by restlessness and delirium. Within a short period of time, we need to evaluate not only the candidates’ cognitive function, but also their compliance and adherence with the instructions on the device managements. Not to mention, life-long education of both patients and care givers after implantable VAD implantation is essential for successful long-term outcomes. However, to determine the indication for DT preoperatively, what matters more is a multidisciplinary and multifaceted preoperative evaluation of device management capabilities. One approach is to have the patients actually hold the HeartMate3 unit, controller, and instruction manual in his or her hands and see how they behave. In some cases, even a simulated brief instructional session can reveal a significant decline in short-term memory capacity. Especially for DT patients, their caregiver might not be physically present with them, 6 months after the implantation. This is clearly indicating that, within a few years, some DT septuagenarians will be managing HeartMate3 at home, living alone. DT is an expensive treatment, but social support is also needed to add up to the high cost of DT. As described below, it is necessary to establish a network that can provide continuous guidance on home care and device management for DT patients, in cooperation with local medical facilities, centered on facilities that perform implantable VAD implantation.

- (3) To prevent complications by improving the quality of care.

For the long-term survival of DT patients, we must not forget to improve the quality of care provided by us to prevent complications, similarly to the BTT population. Complications of implantable VAD are described in textbooks, but according to J-MACS statistics [1] from June 2010 to December 2022, the leading cause of death was cerebrovascular accident (CVA, 34%), and the second leading cause was infection (22%). To achieve long-term survival of more than 5 years in elderly DT patients, it is essential to prevent these lethal complications. The management of aortic regurgitation is also essential for the long-term survival, but this has been covered in another article and is, therefore, omitted from this report [11].

First, I will discuss CVA. HeartMate3 used for DT in Japan is a fully magnetic levitation device with a wide blood-flow pathway around the impeller, which is known to have very high hemocompatibility [12]. Conventional implantable VADs tend to have higher shear stress on the blood due to contact bearings and narrow blood-flow pathways, resulting in blood damage and decreased von Willebrand factor (vWF) activity. Blood damage is thought to be associated with pump thrombosis and CVA, while reduced vWF activity is associated with gastrointestinal bleeding. In fact, a comparison of patients with HeartMate 3 with those with HeartMate II showed a relatively mild decrease in vWF activity in the case of HeartMate 3 [13] and a lower frequency of the hemocompatibility-related complications [14]. In the future, it is expected that post-implant death by CVA will be significantly reduced because HeartMate 3 will be the major component in the DT cohort in Japan.

To further reduce the death by CVA, prevention, early detection, and early treatment are other key features. Prevention of CVA immediately after the implant depends largely on surgical procedures such as meticulous de-airing. For CVA prevention in the chronic phase, appropriate anticoagulation and blood pressure control are mandatory, which depend on the patient's compliance and ability to self-manage. From this perspective, it is important to assess self-management ability before implantation, as described in the previous section. Early detection of CVA in DT septuagenarians living alone is not easy. Remote monitoring should be introduced in cooperation with local medical facilities. For example, Numan et al. reported the utility of combined data from different sources to further improve the prediction performance of algorithms that can be used to remotely monitor VAD patients [15]. Although there is no established protocol for the initial response to the development of CVA in implantable VAD patients, early detection and treatment will surely improve the functional prognosis and ultimately their quality of life.

Our group works closely with neurosurgeons and aims to re-perfuse CVA patients by catheterization within a few hours of stroke onset. If symptoms persist at the time of the first report, we do not hesitate to call and activate a catheter team, as soon as possible, and to perform a CT angiography. If indicated, endovascular treatment is performed promptly. In the case of gross cerebral hemorrhage, urgent craniotomy may be necessary. Since HeartMate3 used in DT is considered more hemocompatible than conventional devices, we generally consider full reversal of antithrombotic therapy with prothrombin complex preparations, fresh-frozen plasma, vitamin K, and platelet concentrate administration.

Next, we discuss infection. In contrast to CVA, the introduction of HeartMate3 did not decrease the frequency of infections. According to the analysis of the MOMENTUM 3 trial by Patel et al., the infection-free rate at 2 years after implantation was only about 36%, which was almost the same as the results for HeartMate 3 and HeartMate II [16]. Infection is also associated with the development of cerebrovascular disease; Zijderhand et al. reported that the hazard ratio for CVA increased approximately twofold for mediastinitis and bloodstream infection after implantable VAD surgery and approximately 1.5-fold for device and driveline infection [17].

To prevent driveline infections, it is important to thoroughly educate patients about wound disinfection technique. However unfortunately, in elderly DT patients and their older caregivers, what they once learned might gradually fade away from their memory. From this point of view, evaluation of self-management ability by a multidisciplinary team before implantation is essential. In addition, our team must continuously confirm that the patient understands the disinfection and management methods appropriately during the regular monthly visits. In DT, where the goal is not HT, how to control infection of the device itself is a significant concern.

As of December 2023, only HeartMate 3 is available for DT indication, and even in the case of pump infection, it cannot be changed to other devices. If tolerated, HeartMate 3 replacement through a re-median sternotomy plus omentopexy should be considered. This is an invasive procedure for DT patients and their quality of life might go down to some degree. If the patient is intolerant to surgery, local drainage is an option. It would take some time for the infection to reach the pump pocket from the driveline exit site. Attempts should be made to lengthen this time. In both BTT and DT cases, our team intentionally lengthen the driveline pathway through three skin incisions in the abdomen (triple tunnel method), so that it would take time for the driveline infection to reach the pump pocket [18]. Although this procedure might be beneficial in preventing lethal pump infections, apparently there is no randomized study or risk-adjusted analysis to prove it. Decisions on such a technical tip to minimize VAD infections should be made by institutional basis at this moment.

Finally, right heart failure (RHF) is another significant issue. From INTERMACS registry, Kapelios et al. reported that the rate of early RHF was 24%, and late RHF (de novo RHF at 6 months or later) was persistent, easy to recur, and its 3-year survival was only 51% (while 73% in no-RHF group) [19]. In contrast to their reports, J-MACS statistical analysis demonstrated that 5-year RHF-free rate was around 80% [1], probably thanks to our careful patient selection. Although DT criteria in Japan precludes the candidates with high risk of RHF after the implantation [7], accurate preoperative prediction of postoperative RHF is still challenging as previously reported [20], and more importantly, since our ultimate goal of DT-VAD support duration is now longer than 10 years, the cumulative incidence of RHF during such a long-term support might not be so low as expected. Although preoperative patient's management and postoperative care for early and late-onset RHF are known to be all challenging [21], we may still have something to do. As for late RHF prediction, our group recently reported the utility of hemodynamic assessment with saline loading to evaluate the right ventricular pre-load reserve function, to reveal that the low right ventricular stroke work index change with saline loading could be a risk factor for late LRHF [22]. In old DT patients, how to deal with severe refractory RHF is a huge problem in Japan, where the option of percutaneous right VAD (RVAD) is limited. As a case report of a BTT patient, we previously reported the surgical technique of minimally invasive RVAD via left thoracotomy to avoid redo-sternotomy RVAD [23]. In the future, we would probably need to combine such a less-invasive approach, even for DT patients, with small and light portable RVAD systems, to develop and implement home RVAD therapy. Thus, to achieve long-term survival with good quality of life in the elderly DT population, similarly to BTT, it is mandatory for us to prevent these untoward complications by improving the quality of care.

- (4) To expand the number of facilities that can take care of DT patients.

As of April 2023, we have 45 BTT-VAD implant institutions, and among them, as of July 2023, 19 institutions are also approved at DT-VAD implant institutions. And as for VAD management institutions, as of January 2024, we have 28 institutions in Japan. Regarding the ideal number of VAD implant and management institutions in Japan could be still controversial, and we would need to the following three issues into consideration. First, regional discrepancy, especially in DT-VAD implantation should be minimized, and probably we should have more DT-VAD implant centers nation widely. Second, the number of VAD management hospital should be expanded, since the number of solitary-living old DT patients, without caregiver, will surely increase as the time goes by, since that is what we are aiming for. However, once irreversible complications happen, he or she would probably not be able to go home alone. To increase VAD management hospital, of course incentive for the management institution providing in-hospital VAD care should be guaranteed. And third, there is always a trade-off between the number and the quality, and too many VAD hospitals with low quality of care does not help at all. To avoid this, inter-institutional collaborations and educational staff exchange programs would be necessary.

- (5) To reduce the burden of caregivers by utilizing social resources.

Although the DT criteria require at least one caregiver who can live with the patient for 6 months after first discharge, ideally caregivers or public services should continue their nursing care after the 6 months (Table 1). Additionally, in the criteria for safe management at home care, it is documented that caregiver must continue providing psychological and economic support for the patients, even after the 6 months, and need to accompany with them, especially when they go far away including for hospital appointments. Although we do not have much data on the psychological burden of caregiver for DT patients in Japan, it is apparent that it does exist, although a validated measure of caregiver burden is still limited [24]. Home visiting nursing to replace the caregiver work, despite temporarily, could be a solution. However, even with the insurance coverage, home visiting nursing is quite expensive, especially when primary diseases of DT patients are ischemic cardiomyopathy, not dilated one, and they are not eligible for the benefits of intractable disease. To lighten the burden of such a caregiver, we VAD providers must speak up to enhance socioeconomic support for ischemic DT families. Another essential countermeasure to release caregivers is to keep a good quality of life in old DT patients to make them independent forever. Our goal is not just making them live long but making them even much happier. For this aim, it is crucial to help them to find a purpose of life and to keep activities of daily living, such as employment, schooling, and participation in social activities, just like the general elderly population. In fact, Kugler et al. investigated the association between re-employment after VAD implantation and health-related quality of life, encouraging clinicians to support professional employment for selected VAD patients [25]. Their patients were mostly BTT, but such efforts on DT-VAD patients could also lead to the improvement of patients’ quality of life and eventually bring about the reduction in caregiver obligation.

- (6) To establish a home palliative care system based on advance care planning.

Finally, how to make them end their own lives, as they wish, is another indispensable consideration. We must profoundly discuss advance care planning, with the patients and caregivers before VAD implantation, ideally when they both are completely healthy. In Japan, unfortunately, VAD-specialized palliative care team is extremely rare [26]. Therefore, surgeons, cardiologists, VAD coordinators, and other co-medicals are required to take the role by multidisciplinary approach. Occasionally, it may take years to establish a relationship with patients and caregivers, to eventually find out what their life goal is. In Japan, where deactivation of VAD is not legally easy [27], some patients would prefer to take home palliative care. In the current setting, a VAD patient is required to visit us at least once a month to receive insurance reimbursement for out-patient care, this rule needs to be modified in the future to make them spend more peaceful end-of-life time at home with family members or caregivers.

## Conclusion

In the present manuscript, we discussed six points to maintain a safe and high quality of life during long-term support in DT patients in Japan. Prevention and management of remote complications are the key features for this goal, but it is also mandatory to maintain device management skills through both patient and caregiver life-long education as well. The number of DT who are elderly but have limited family support is expected to increase in the future. Although there are certain man-power limitations among us, we must keep making effort on what we can do. In addition to the very best care by individual medical teams for the patients they are facing, it is also essential to establish a national system to follow up DT population within local medical regions. We, the DT medical team, must keep working on awareness campaign to the public regarding DT in Japan to ensure that DT patients are widely accepted by society.

## Data Availability

The authors confirm that the data supporting the findings of this study are available within the article.
